# Heterogeneity of Ara h Component-Specific CD4 T Cell Responses in Peanut-Allergic Subjects

**DOI:** 10.3389/fimmu.2018.01408

**Published:** 2018-06-25

**Authors:** Amedee Renand, Marry Farrington, Elizabeth Whalen, Erik Wambre, Veronique Bajzik, Sharon Chinthrajah, Kari C. Nadeau, William W. Kwok

**Affiliations:** ^1^Benaroya Research Institute at Virginia Mason, Seattle, WA, United States; ^2^Virginia Mason Medical Center, Seattle, WA, United States; ^3^Sean N. Parker Center for Allergy Research at Stanford University, Division of Pulmonary and Critical Care Medicine, Stanford University School of Medicine, Stanford, CA, United States; ^4^Division of Allergy, Immunology and Rheumatology, Stanford University School of Medicine, Stanford, CA, United States; ^5^Department of Medicine, University of Washington, Seattle, WA, United States

**Keywords:** Ara h, peanut allergy, TH2 cell, CRTH2, IgE, class II tetramer, CD154 assay

## Abstract

Understanding the peanut-specific CD4 T cell responses in peanut-allergic (PA) subjects should provide new insights into the development of innovative immunotherapies for the treatment of peanut allergy. Although peanut-specific CD4 T cells have a TH2 profile in PA subjects, the immunogenicity of different Ara h components in eliciting specific CD4 T cell responses and the heterogeneity of these Ara h-reactive TH2 cells remains unclear. In this study, we investigated Ara h 1, 2, 3, 6, and 8-specific T cell responses in PA and sensitized non-peanut-allergic (sNPA) subjects, using the CD154 upregulation assay and the class II tetramer technology. In the PA group, T cells directed against Ara h 1, 2, 3, and 6 have a heterogeneous TH2 phenotype characterized by differential expression of CRTH2, CD27, and CCR6. Reactivity toward these different components was also distinct for each PA subject. Two dominant Ara h 2 epitopes associated with DR1501 and DR0901 were also identified. Frequencies of Ara h-specific T cell responses were also linked to the peanut specific-IgE level. Conversely, low peanut-IgE level in sNPA subjects was associated with a weak or an absence of the allergen-specific T cell reactivity. Ara h 8-specific T cell reactivity was weak in both PA and sNPA subjects. Thus, peanut-IgE level was associated with a heterogeneous Ara h (but not Ara h 8)-specific T cell reactivity only in PA patients. This suggests an important immunogenicity of each Ara h 1, 2, 3, and 6 in inducing peanut allergy. Targeting Ara h 1-, 2-, 3-, and 6-specific effector-TH2 cells can be the future way to treat peanut allergy.

## Introduction

Peanut allergy is an important health issue as it affects approximately 2% of children and can lead to potentially fatal anaphylactic reaction (1). Currently, there is no approved method for treatment other than strict avoidance. Although introduction of peanut into the diet of high risk infants appears to be a promising strategy in limiting peanut allergy progression (2), a better understanding of the immune response against peanut allergen can lead to development of an innovative therapeutic strategy to treat peanut-allergic (PA) subjects.

Correct diagnosis of peanut allergy has been difficult in clinical practice. Diagnosis is complicated by the observation that subjects with peanut-specific IgE and or a positive skin prick test result can be non-reactive to peanut food challenge and can consume peanut safely. Oral food challenge, which is time consuming and poses a risk to the subject, has been considered as the only option for accurate diagnosis. More recently, it has been demonstrated that Ara h component-specific IgE test and basophil activation test may be useful to distinguish between allergic and tolerant subjects, without performing oral food challenges. At this level, Ara h 2-IgE level and Ara h 2 basophil responses seem to be the better predictor of an allergic response, and mono-sensitization to Ara h 8 is linked to tolerance (3–7). But the link between peanut-specific IgE with the specific T cell reactivity is unknown.

Type 2 helper T cells are required for Ig switching to generate IgE memory B cells and plasma cells, and play an important role in the immune cascade that leads to food allergy (8). Targeting these helper T cells is a viable option for effective antigen-specific immunotherapy (9–11). However, Ara h component-specific TH2 cell responses and the heterogeneity of these TH2 cells are not well characterized within PA subjects (12). The relative influence of each Ara h component in eliciting specific T cell responses has not been studied. In addition, the link between the reactivity of Ara h component-specific CD4 T cell populations and an allergic or non-allergic response in sensitized subjects is unclear (12–19). We hypothesize that Ara h component-specific T cell responses could be linked to the peanut-IgE level. In this study, we investigated Ara h-specific T cell responses against Ara h 1, 2, 3, 6, and 8 (with Ara h 1, 3, and 6 as a pooled response) using pools of peptides covering the aforementioned Ara h allergens in both PA and sensitized non-peanut-allergic (sNPA) subjects (Tables 1 and 2). Phenotypes of these Ara h-reactive T cells were also being evaluated. In addition, correlation between Ara h component-specific T cells and Ara h component-specific IgE was examined in both groups. Ara h-specific TH2 responses were found to be linked to an allergic response and to the peanut-IgE level. Conversely, a weak or an absence of specific T cell response was observed in non-allergic but sensitized patients. Technically, a novel T cell epitope mapping approach was developed by combining the CD154 upregulation assay and tetramer assay, DR0901 restricted and DR1501 restricted Ara h 2 epitopes were identified.

**Table 1:** Patients’ cohort.

Samples	Age	Sex	Allergic status	HLA-DRB1	SPT	IgE (kU/L)								Reaction history
						Peanut	Ara h2	Ara h1	Ara h3	Ara h8	Timothy	Alder	Birch	
A1	11	M	Allergic	15:01/15:02	15	100	100	80.2	28.6	0.34	1.35	1.34	1.07	Ingestion hx
A2	19	M	Allergic	13:01/04	8	10.8	7.38	0.17	0.12	5.08	1.68	100	100	Ingestion hx
A3	8	M	Allergic	04:01/09:01	30	100	100	18.4	7.46	<0.1	<0.35	<0.35	<0.35	Ingestion hx
A4	10	M	Allergic	04:01/09:01	ND	100	61.7	35.6	2.61	3.19	10.3	82.4	22.3	DBPCFC
A5	12	M	Allergic	08.01/09.01	20	20.7	16.3	6.24	0.25	0.26	1.89	3.69	1.11	DBPCFC
A6	8	M	Allergic	07:01/12:01	ND	100	7.66	56	100	53.6	71.5	100	100	Ingestion hx
A7	21	M	Allergic	15:01/03:01	20	76.6	51.4	32.4	11.4	31.5	50.5	100	97.8	DBPCFC
A8	10	F	Allergic	03:01/07:01	20	84.6	50.9	36.7	7.87	1.42	17.2	12.8	12.3	Ingestion hx
A9	21	F	Allergic	11:01/09:01	20	68.3	41	9.8	5.43	3.18	18.5	14.3	12.8	Ingestion hx
A10	12	F	Allergic	03:01/11:04	10	47.7	33.9	19.5	2.55	2.11	0.77	7.12	5.57	Ingestion hx
A11	7	F	Allergic	03:01/03:07	10	5.43	5.77	0.44	0.14	<0.1	88.9	<0.35	<0.35	Open challenge
A12	12	F	Allergic	04:01/13:01	12	1.88	1.64	<0.1	<0.1	<0.1	29.7	<0.35	<0.35	Ingestion hx
A13	11	M	Allergic	07:01/13:01	5	100	100	100	37.6	<0.1	<0.35	<0.35	<0.35	Open challenge
A14	17	F	Allergic	15:01/13:01	9	100	47.3	78.9	12.2	0.36	1.01	2.55	2.05	Ingestion hx
N1	11	F	Non-allergic	01:01/08:01	ND	13.4	<0.1	<0.1	<0.1	19.1	35.8	69.2	ND	Ingests peanut
N2	16	F	Non-allergic	11:01/13:02	1	1.76	<0.1	<0.1	<0.1	<0.1	100	0.4	<0.35	Ingests peanut
N3	9	F	Non-allergic	07:01/11:01	0	7.14	<0.1	<0.1	<0.1	2.7	100	17	ND	Ingests peanut
N4	13	M	Non-allergic	ND	2	1.42	<0.1	<0.1	0.24	<0.1	<0.35	<0.35	<0.35	Ingests peanut
N5	10	M	Non-allergic	ND	19	0.47	0.11	<0.1	<0.1	<0.1	10.1	<0.35	<0.35	Pass challenge (2,400 mg)
N6	10	M	Non-allergic	ND	8	1.92	0.13	<0.1	<0.1	0.13	31.4	3.68	3.25	Pass challenge (2,400 mg)

**Table 2:** Patients’ allergic manifestations.

Samples	Allergic status	Clinical information
A1	Allergic	Generalized urticaria, rhinitis
A2	Allergic	Generalized urticaria, nausea/vomiting
A3	Allergic	Generalized urticaria, nausea/vomiting
A4	Allergic	Generalized urticaria, nausea/vomiting, wheezing, rhinitis, treated with epinephrine
A5	Allergic	Generalized urticaria
A6	Allergic	Generalized urticaria, nausea/vomiting, wheezing, rhinitis, treated with epinephrine
A7	Allergic	Stomach pain, vomiting, treated with epinephrine
A8	Allergic	Generalized urticaria
A9	Allergic	Generalized urticaria, wheezing, treated with epinephrine
A10	Allergic	Generalized urticaria
A11	Allergic	Generalized urticaria
A12	Allergic	Generalized urticaria, wheezing
A13	Allergic	Generalized urticaria, rhinitis
A14	Allergic	Stomach pain/nausea, throat swelling
N1	Non-allergic	Walnut allergy/ingests peanut
N2	Non-allergic	Tree nut allergy/ingests peanut
N3	Non-allergic	Cashew allergy/ingests peanut
N4	Non-allergic	Tree nut allergy/ingests peanut
N5	Non-allergic	Pass challenge (2,400 mg)
N6	Non-allergic	Tree nut allergy/pass challenge (2,400 mg)

## Materials and Methods

### Subjects

A total of 20 subjects with a median age of 11 (7–22 years) were recruited from the Virginia Mason Medical Center Allergy Clinic, Benaroya Research Institute, and Stanford University School of Medicine. All subjects were HLA typed by using sequence-specific oligonucleotide primers with Unitray SSP Kits (Invitrogen, CA, USA) and tested positive after skin prick test (>4 mm) and/or positive for peanut-IgE (>1 kU/L). Fourteen of those subjects (7–22 years, median age = 11.5 years) had peanut allergy diagnosed by an allergy physician based on peanut-specific IgE and good clinical history of peanut-induced reaction (9/14), open peanut challenge (2/14), or DBPCFC to peanut (3/14) (Tables 1 and 2). Six sensitized subjects (9–16 years, median age = 10.5 years) with peanut IgE > 0.35 kU/L were recruited as sNPA because they passed a peanut oral challenge (4,000 mg) or could consume peanut ad lib without signs or symptoms of an allergic reaction. ImmunoCAP scores for peanut-, Ara h component-, Alder-, Timothy grass- and Birch-IgE were obtained (Phadia AB) (Table 1). All subjects were recruited with informed consent and institutional review board approval.

### *Ex Vivo* Analysis of Peanut Allergen-Specific CD4^+^ T Cells

For the CD154 (CD40L) expression assay, 10 to 20 × 10^6^ peripheral blood mononuclear cells (PBMCs) (at a final concentration of 10 × 10^6^/mL) were stimulated for 3 h at 37°C with 5 µg/mL of synthesized peptide pools (20 amino acids in length with a 12 amino acid overlap; Mimotopes, Australia) spanning all of the Ara h 2, 1, 3, 6, and 8 sequences [Ara h 2 (p1–p20), Ara h 1 (p1–p74), Ara h 3 (p1–p62), Ara h 6 (p1–p14), and Ara h 8 (p1–p19)] in 10% human serum RPMI medium in the presence of 1 µg/mL anti-CD40 (HB14, Miltenyi Biotec). After 3 h of specific peptide stimulation, PBMCs were first labeled with PE-conjugated CD154 and CD154^+^ cells and then enriched using anti-PE magnetic beads (Miltenyi Biotec). A 1/10th fraction of unenriched cells was saved for analysis for frequency determination. Frequency was calculated by using the formula *F* = *n*/*N*, where *n* designates the number of CD154-positive cells in the bound fraction after enrichment and *N* is the total number of CD4^+^ T cells (calculated as 10× the number of CD4^+^ T cells in 1/10th unenriched fraction that was saved for analysis). After enrichment, cells were stained with PerCP-Cy5.5 anti-CD14 (HCD14, BioLegend), PerCP-Cy5.5 anti-CD19 (HIB19, BioLegend), V500 anti-CD4 (RPA-T4, BD Biosciences), Alexa Fluor 700 anti-CD45RA (HI100, BD Biosciences), PE-Cy7 anti-CD194 (TG6/CCR4, BioLegend), Alexa Fluor 647 anti-CD294 (CRTH2, BM16, BD Biosciences), APC-Cy7 anti-CD27 (O323, BioLegend), Brilliant Violet 421 anti-CD196 (CCR6, 11A9, BD Biosciences) antibodies, and Cell viability solution (BD Via-Probe, BD Biosciences). Staining with HLA-DRB1*0901/Ara h 2_30–49_ and HLA-DRB1*1501/Ara h 2_89–108_ tetramers was carried out as previously described (20, 21).

### “Modified” CD154 Upregulation Assay for Epitope Mapping

A maximum of 100 CD154^+^CD45RA^−^CD4^+^ T cells were sorted per well (U-bottom 96-well plate) after the CD154 expression assay and expended in the presence of 1.5 × 10^5^ autologous irradiated PBMCs, 1 µg/mL PHA (Sigma), human IL-2 (10 U/mL, Roche), and T cell growth media. After 10–14 days, cells were transferred to a flat bottom 48-well plate and restimulated with irradiated PBMCs, 1 µg/mL PHA (Sigma), human IL-2 (10 U/mL, Roche), and T cell media. Cells were split and fed as appropriate. Once the cells were successful expanded, epitope mapping experiments were performed. For mapping, 10^5^ expanded T cells were stimulated for 3 h at 37°C with 5 µg/mL of synthesized Ara h 2 peptide pools (Ara h 2 peptides were divided into four pools with five peptides per pool) in 96-well plate in the presence of 1 µg/mL anti-CD40 (HB14, Miltenyi Biotec) and 10^5^ autologous PBMCs, in 10% human serum RPMI medium. After 3 h, cells were stained with PE-CD154 and APC-CD4 antibodies. Pool giving a positive response was retested with 40 µg/mL blocking antibodies anti-HLA-DR (L243) or anti-HLA-DQ (SPVL3) to examine DR or DQ restriction. Peptides from pool giving a positive response were then tested with individual peptides from the positive pool. Individual identified peptides (epitope) were loaded into the biotinylated HLA-DR or HLA-DQ proteins to generate tetramers for staining as described (22).

### Intracellular Cytokine Staining

*In vitro* intracellular cytokine staining combined with MHC class II tetramer staining was performed, as previously described (23). For *in vitro* intracellular cytokine staining, cells were stained with the corresponding PE-labeled tetramers for 60 min at 37°C. Cells were then restimulated with 25 ng/mL phorbol 12-myristate 13-acetate and 1 mg/mL ionomycin in the presence of 10 mg/mL Brefeldin-A for 4 h at 37°C. Cells were then stained with APC-Cy7 anti-CD4 (OKT3, BioLegend), Alexa Fluor 488 anti-IL-4 (8D4-8, eBioscience), Alexa Fluor 700 anti-IFN-γ (4S.B3, BioLegend), APC anti-IL5 (TRFK5, BD Biosciences), PerCP-Cy5.5 anti-IL-17 (BL168, BioLegend), PE-Cy7 anti-IL-10 (JES3-9D7, BioLegend) antibodies, and fixable viability stain 450 (BD Biosciences).

### Statistical Analysis

Statistical analysis was performed using Prism 5.0 software (GraphPad) or using R software for the ROC curve analysis. Cutoff values were determined by using the predictive score giving the better/closer sensitivity and specificity percentage after the ROC curve analysis. Data were compared using Mann–Whitney test or using Kruskal–Wallis test and Dunn’s Multiple Comparison Test (*p ≤ 0.05, **p ≤ 0.01, and ***p < 0.0001). For multiple comparison tests, *p* values were adjusted according to Bonferroni–Holm test.

## Results

### CD154 Assay Reveal Ara h-Specific T Cell Responses Linked to an Effector-TH2 Phenotype in PA Subjects

**Figure 1:**
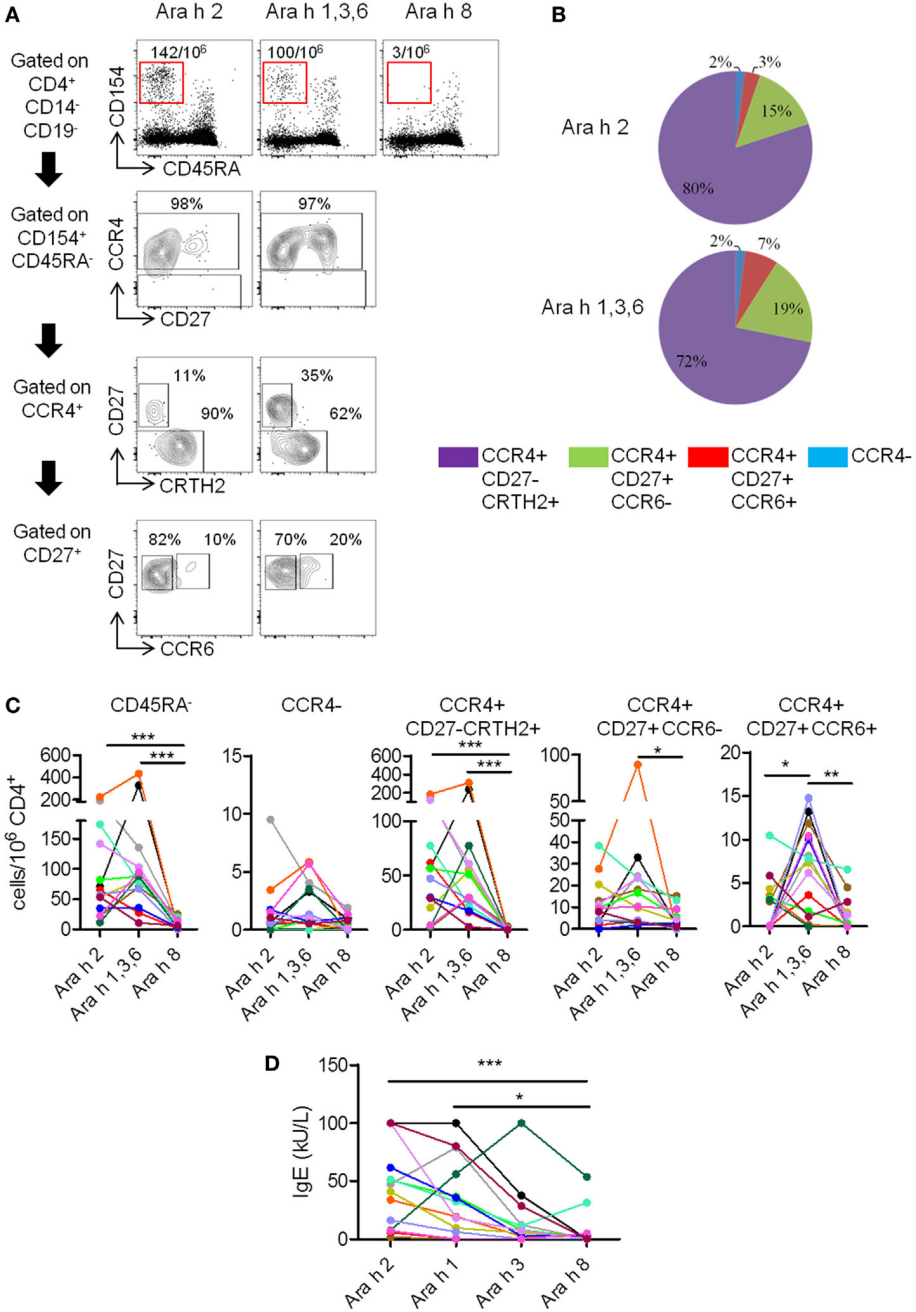
T cell and IgE responses against major Ara h components in peanut-allergic (PA) subjects. **(A)** CCR4, CD27, CRTH2, and CCR6 expression on CD4^+^CD45RA^−^CD154^+^ T cells after Ara h peptide stimulation. Frequency per 10^6^ CD4^+^ T cells and percentage werw indicated. **(B)** Percentage repartition of CCR4^+^CD27^−^CRTH2^+^, CCR4^+^CD27^+^CCR6^−^, CCR4^+^CD27^+^CCR6^+^, and CCR4^−^ T cell subsets within CD4^+^CD45RA^−^CD154^+^ T cells after Ara h peptide stimulation. **(C)** Frequency of CD154^+^CD4^+^ T cell populations after peptide stimulation in PA subjects (*n* = 14). **(D)** Specific-IgE levels in allergic subjects. For panels **(C,D)**, each PA subject was represented by a distinct color. Statistical analysis was performed using Kruskal–Wallis test and Dunn’s Multiple Comparison Test (*p ≤ 0.05, **p ≤ 0.01, and ***p < 0.0001). For multiple comparison tests, *p* values were adjusted according to Bonferroni–Holm test. For panel **(C)**, *p* value < 0.016 was considered as significant. For panel **(D)**, *p* value < 0.0125 was considered as significant.

Ara h component-specific CD4^+^ T cell responses and IgE responses were examined in PA subjects (Figure 1; Table 1). We focused on Ara h 2 and Ara h 8, as IgE reactivity toward these two components was most effective in discriminating PA allergic from tolerant subjects (1–5). In addition, T cell responses toward Ara h 1, 3, and 6 were also examined as a pooled response because available number of PBMCs was limited (blood from children). For these experiments, specific T cell responses were evaluated against three sets of overlapping peptides derived from Ara h 2, Ara h 8 and a combination of Ara h 1, Ara h 3, and Ara h 6 (Ara h 1, 3, and 6) using CD154 (CD40L) upregulation assays (23–25).

In PA subjects, specific memory (CD45RA^−^) responses were observed after stimulation with Ara h 2 or Ara h 1, 3, and 6 peptides, but absent or weak after stimulation with Ara h 8 peptides (Figures 1A,C). Ara h 2- and Ara h 1, 3, and 6-specific T cells primarily exhibited a phenotype of effector-TH2 cells (CCR4^+^CD27^−^CRTH2^+^) (26–28), but “classical” TH2 phenotype (CCR4^+^CD27^+^CCR6^−^) or TH2/TH17-like phenotype (CCR4^+^CD27^+^CCR6^+^) (24) was also observed (Figures 1A–C). T cell responses toward Ara h 2 and Ara h 1, 3, and 6 were significantly higher compared with Ara h 8. Frequencies of Ara h 1, 3, and 6-specific cells with TH2/TH17-like phenotype were significant higher compared with frequencies of Ara h 2 and Ara h 8-specific cells of similar phenotype (Figure 1C). In addition, different PA subjects have their distinct profile of Ara h component-specific T cell responses. There were two subjects with mainly Ara h 2-specific CD45RA^−^CD4^+^ T cell responses, three subjects with robust Ara h 1, 3, and 6-specific responses and minimal Ara h 2-specific responses, eight subjects with robust Ara h 2-, 1, 3, and 6-specific responses and one subject with only weak responses toward Ara 2, 1, 3, and 6. Effector-TH2, “classical” TH2, and TH2/Th17 responses also varied among different PA subjects (Figure 1C).

IgEs directed against peanut and Ara h components were also analyzed (Figure 1D; Table 1). In PA subjects, Ara h 2 IgE was significantly higher compared with Ara h 8 IgE (Figure 1D).

In summary, specific T cell response in PA subjects was heterogeneous, mainly oriented against Ara h 2 and Ara h 1, 3, and 6 allergens, and associated with an effector-TH2 (CCR4^+^CD27^−^CRTH2^+^) response that dominated over a “classical” TH2 response (CCR4^+^CD27^+^). The TH2/TH17-like phenotype has already been observed in patients with other food allergy but is not present in non-allergic patients, and could be dependent of the nature of the allergen (23, 24). At the IgE level, the response was mainly directed against Ara h 2 and Ara h 1 in PA subjects, as previously described (3).

### Single Ara h Component-Specific Effector-TH2 Responses Correlate With Peanut-Specific IgE Level

We performed correlation analysis between frequency of Ara h 2-, Ara h 1, 3, and 6-, and Ara h 8-specific T cells and IgE levels of different Ara h components (Table 3). Frequencies of Ara h 2- and Ara h 1, 3 and 6-specific memory CCR4^+^ T cells robustly correlated with total peanut (*p* = 0.0025 and *p* = 0.0038), Ara h 2 (*p* = 0.0001 and *p* = 0.0009), Ara h 1 (*p* = 0.0005 and *p* = 0.0008), and Ara h 3 (*p* = 0.0014 and *p* = 0.0007) IgE levels but not with Ara h 8 IgE levels (Table 3). This observation was mainly due to the robust correlation of the frequencies of Ara h 2 and Ara h 1, 3, and 6-specific CCR4^+^CD27^−^CRTH2^+^ effector-TH2 cells with total peanut (*p* = 0.003 and *p* = 0.0022), Ara h 2 (*p* < 0.0001 and *p* = 0.0017), Ara h 1 (*p* = 0.0004 and *p* = 0.0007), and Ara h 3 (*p* = 0.0013 and *p* = 0.0004) IgE levels but not with Ara h 8 IgE levels (Table 3). Frequencies of Ara h 2- and Ara h 1, 3, and 6-specific “classical” TH2 cells and/or TH2/TH17-like cells could also correlate with Ara h 2, Ara h 1, and/or Ara h 3 IgE levels. This observation suggests that “classical” TH2- and TH2/TH17-like cells also participate on the accumulation of IgE but are less effective in comparison with effector-TH2 cells. No correlation was observed between Ara h 8-specific T cells and any of the Ara h components IgE levels (Table 3).

**Table 3:** Spearman correlation analysis between T cell and IgE responses.

			IgE levels									
			Ara h2		Ara h1		Ara h3		Ara h8		Peanut	
			*R*	*P*	*R*	*P*	*R*	*P*	*R*	*P*	*R*	*P*
T cell frequency	CD27^+^CCR6^+^	Ara h 2	0.15	ns	0.39	ns	0.28	ns	0.17	ns	0.00	ns
		Ara h 1, 3, 6	***0.49***	***0.0291***	***0.48***	***0.0327***	0.27	ns	−0.15	ns	0.25	ns
		Ara h 8	0.07	ns	0.07	ns	0.02	ns	−0.02	ns	−0.09	ns

	CD27^+^CCR6^−^	Ara h 2	***0.51***	***0.0227***	0.17	ns	***0.49***	***0.0291***	0.22	ns	0.33	ns
		Ara h 1, 3, 6	***0.57***	***0.0082***	0.31	ns	***0.51***	***0.0222***	−0.12	ns	0.34	ns
		Ara h 8	0.16	ns	−0.12	ns	0.17	ns	0.21	ns	0.13	ns

	CD27^−^CRTH2^+^	Ara h 2	***0.77***	***<0.0001***	***0.71***	***0.0004***	***0.67***	***0.0013***	0.03	ns	***0.63***	***0.003***
		Ara h 1, 3, 6	***0.66***	***0.0017***	***0.69***	***0.0007***	***0.72***	***0.0004***	0.16	ns	***0.64***	***0.0022***
		Ara h 8	−0.03	ns	−0.13	ns	−0.06	ns	−0.24	ns	−0.10	ns

	CCR4^+^	Ara h 2	***0.75***	***0.0001***	***0.71***	***0.0005***	***0.66***	***0.0014***	0.12	ns	***0.64***	***0.0025***
		Ara h 1, 3, 6	***0.68***	***0.0009***	***0.69***	***0.0008***	***0.69***	***0.0007***	0.16	ns	***0.62***	***0.0038***
		Ara h 8	0.25	ns	0.16	ns	0.23	ns	0.20	ns	0.20	ns

	IgE levels	Ara h 2	–	–	***0.88***	***<0.0001***	***0.82***	***<0.0001***	0.11	ns	***0.85***	***<0.0001***
		Ara h 1	–	–	–	–	***0.95***	***<0.0001***	0.26	ns	***0.91***	***<0.0001***
		Ara h 3	–	–	–	–	–	–	0.26	ns	***0.87***	***<0.0001***
		Ara h 8	–	–	–	–	–	–	–	–	0.39	ns

*R = Spearman r; P = P value*.

*Bold italic font indicates P < 0.05*.

We also noted a high correlation between peanut and Ara h 2 and Ara h 1 and Ara h 3 IgE levels. However, they did not correlate with Ara h 8 IgE level.

### Validation of the CD154 Assay by Using the Class II Tetramer Technology to Characterized Ara h 2-Specific T Cell Responses

**Figure 2:**
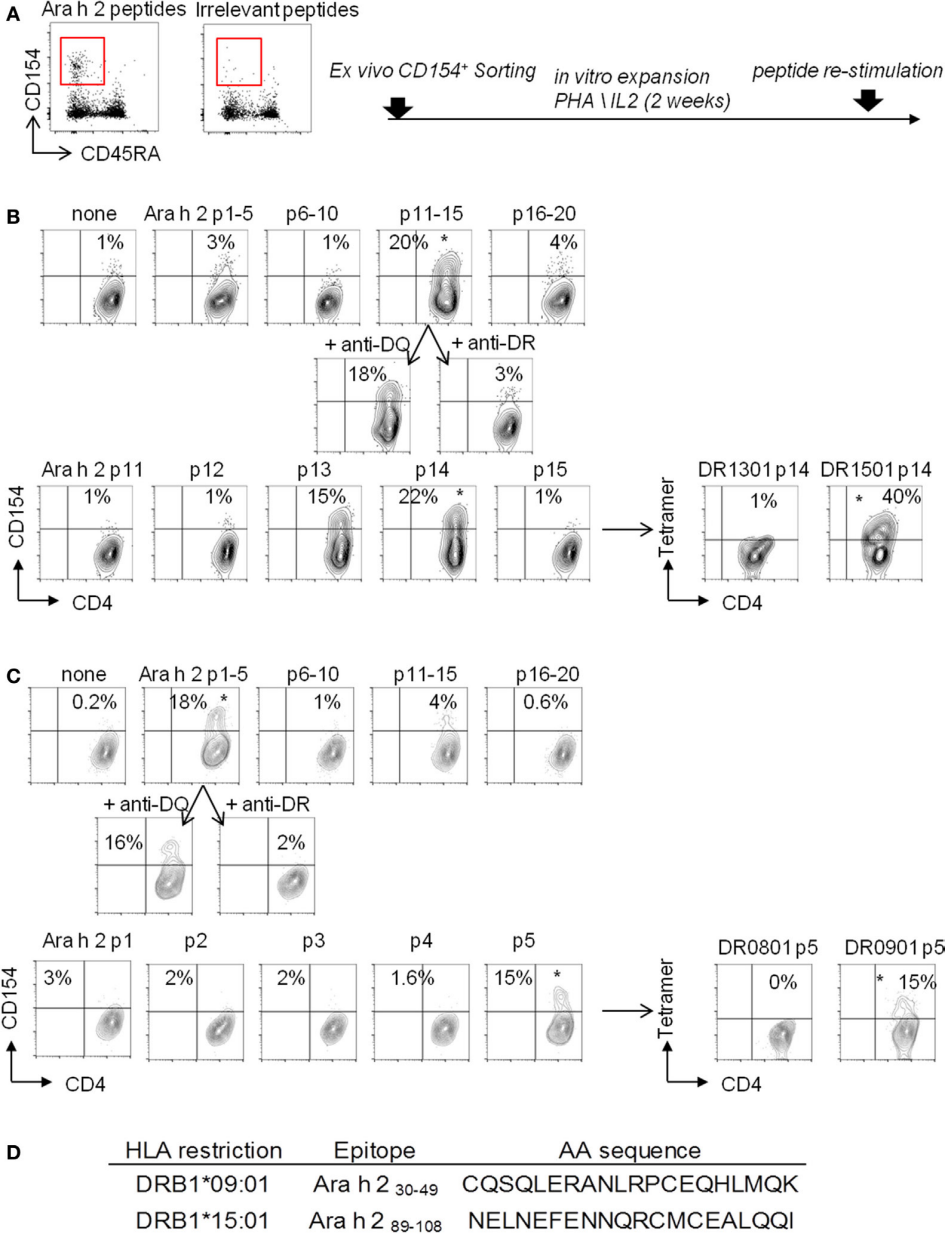
“Modified” CD154 upregulation T-cell epitope mapping. CD154^+^CD45RA^−^CD4^+^ T cells were sorted **(A)** and expended in presence of autologous-irradiated peripheral blood mononuclear cells (PBMCs), 1 µg/mL PHA, and human IL-2 **(B)**. **(B,C)** For epitope mapping, 10^5^ cells were stimulated for 3 h with 5 µg/mL of synthesized Ara h 2 peptide pools with autologous PBMCs. Pool giving a positive response in the CD154 assay was retested with 40 µg/mL blocking antibodies anti-HLA-DR or anti-HLA-DQ. Peptides from pool giving a positive response were then tested individually as descried before. Then, individual peptides, giving a positive response, were loaded into the biotinylated HLA-DR protein to generate tetramers and tested *in vitro*. **(D)** Table of Ara h 2 T cell epitopes identified.

Of the 14 PA subjects in our cohort who had a TH2 cell reactivity, 7 subjects expressed the HLA-DRB1*09:01 (subjects 3, 4, 5, and 9) or *15:01 (subjects 1, 7, and 14) (Table 1), which represented 50% of this group. We performed a “modified” CD154 upregulation T cell epitope mapping for Ara h 2, based on the sorting and the expansion of CD154^+^-reactive T cells after the CD154-upregulation assay (Figure 2). We identified two dominant Ara h 2 epitopes, Ara h 2_31–50_ and Ara h 2_91–110_, restricted to the HLA-DRB1*901 and HLA-DRB1*1501, respectively, as confirmed by tetramer staining (Figure 2).

**Figure 3:**
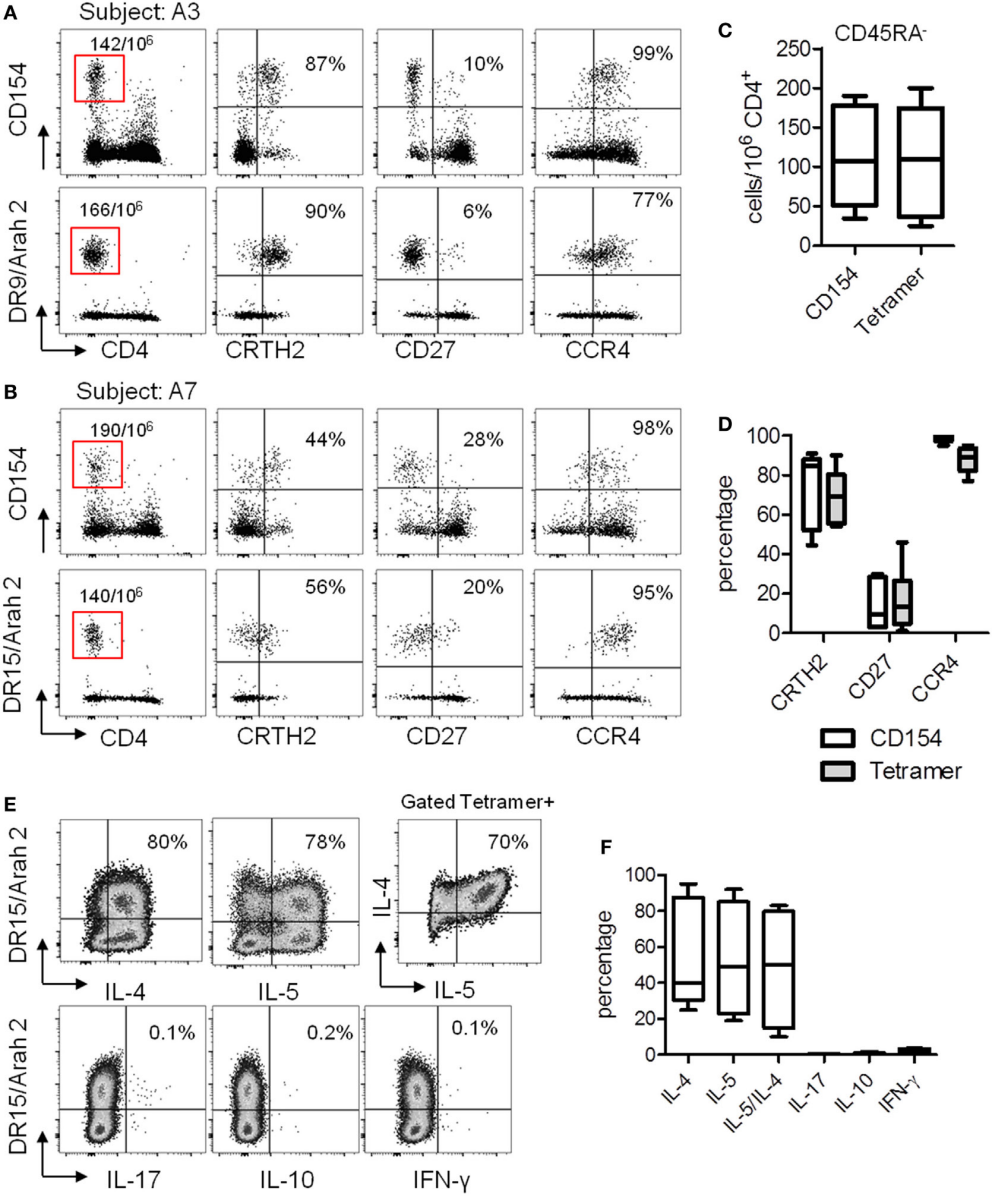
T cell responses against Ara h 2 using both CD154 expression assay and class II tetramer. **(A,B)** CCR4, CD27, and CRTH2 expression on CD4^+^CD45RA^−^CD154^+^ T cells after Ara h peptide stimulation or using class II tetramer loaded with the epitope identified by “modified” tetramer-guided epitope mapping in an HLA DR9 **(A)** and an HLA DR15 **(B)** allergic subject. Frequency per 10^6^ CD4^+^ T cells and percentage were indicated. **(C)** Frequency of CD154^+^CD4^+^ T cell populations after peptide stimulation or using class II tetramer in peanut-allergic subjects (*n* = 7). **(D)** Percentage of CRTH2, CD27, and CCR4 on Ara h 2-specific CD4^+^ T cells after Ara h 2 peptide stimulation or after class II tetramer staining. **(E)** Cytokines intracellular staining on cultured Ara h 2-specific T cells from an HLA DR15 allergic child. **(F)** Percentage of IL-4, IL-5, IL-17, IL-10, and IFN-γ on Ara h 2-specific CD4^+^ T cells after class II tetramer staining.

We next performed direct *ex vivo* class II tetramer staining in these seven PA subjects and compared the data to the results obtained by using the CD154 assay. As we previously observed (23, 25), the CD154 assay gave similar results compared with class II tetramer technology (Figures 3A–D). Indeed, using these two different methods, we observed equal frequency of memory Ara h 2-specific CD4 T cells in PA subjects (Figure 3C). Moreover, regarding the phenotype of these memory Ara h 2-specific CD4 T cells, we did not observe significant divergence between the CD154 assay and the Class II tetramer technology. Thus we confirmed that the Ara h 2-reactive CD4 T cell had an effector-TH2 cell phenotype (Figure 3D). Ara h 2-specific cell lines were established. Cytokine profile analysis shows that these specific T cells produced IL-4 and IL-5 but not IFN-γ or IL-17 (Figures 3E,F).

### sNPA Subjects Had Very Low Specific T Cell Response Against Ara h Components

**Figure 4:**
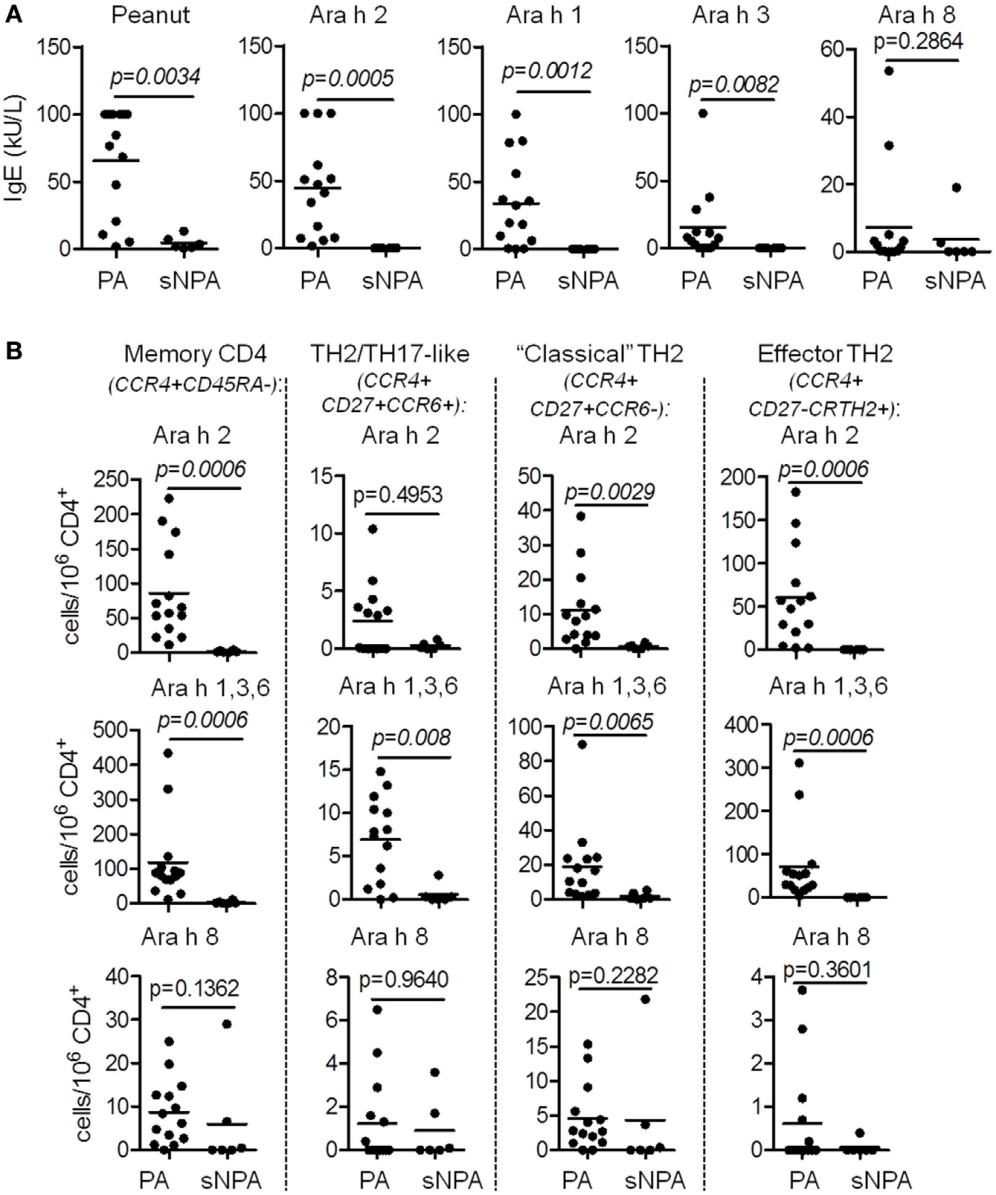
T cell and IgE responses against major Ara h components on sensitized non-peanut-allergic (sNPA) subjects. **(A)** Specific-IgE levels on peanut-allergic (PA; *n* = 14) and sNPA (*n* = 6). **(B)** Frequency of CD154^+^CD4^+^ populations (CD45RA^−^; CCR4^+^CD27^−^CRTH2^+^; CCR4^+^CD27^+^CCR6^−^, and CCR4^+^CD27^+^CCR6^+^) after Ara h peptide stimulation on PA (*n* = 14) and sNPA (*n* = 6) subjects.

**Figure 5:**
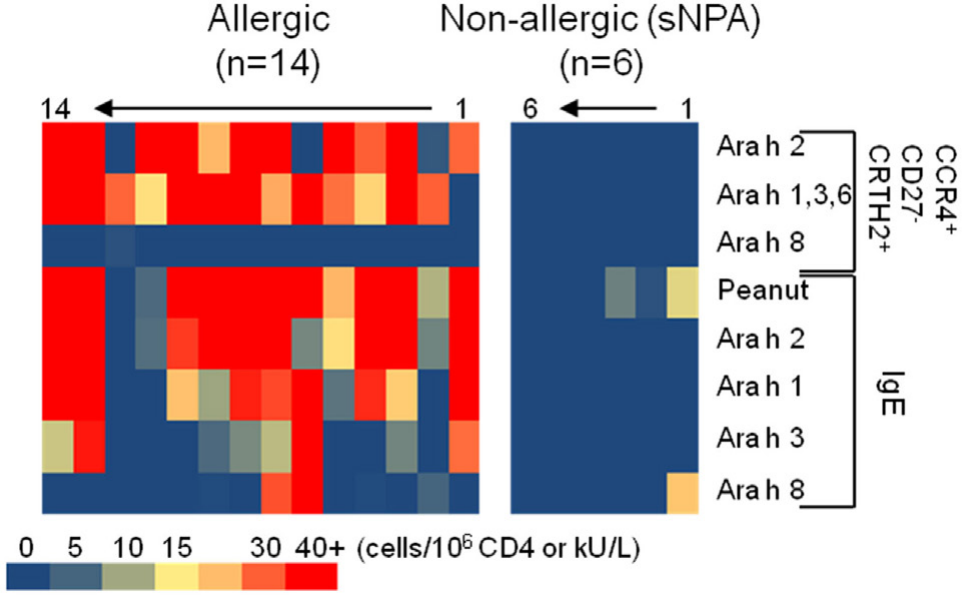
Heat map representation of CD154^+^CD4^+^ T cell populations’ frequencies and IgE levels on peanut-allergic and sensitized non-peanut-allergic subjects.

We investigated Ara h-specific T cell responses and Ara h-IgE levels in six sNPA subjects with low sensitization and compared the results with those obtained from PA subjects (Figures 4 and 5; Tables 1 and 2). Sensitized NPA present low peanut sensitization (Tables 1 and 2) but are not allergic to peanut and, thus constitute a well control group. Sensitized NPA subjects presented significantly less IgE directed against peanut extract, Ara h 2, Ara h 1, and Ara h 3, compared with PA subjects (Figure 4A). However, the level of Ara h 8 IgE was low and not significantly different between the two groups (Figure 4A). Similar outcomes were also observed for specific T cells. Sensitized NPA subjects presented significantly lower frequencies of Ara h 2- or Ara h 1, 3, and 6-specific “classical” TH2 cells and effector-TH2 cells compared with PA subjects. With respect to the TH2/TH17-like subset, significant difference was only observed with the frequencies of Ara h 1, 3, and 6-specific cells (Figure 4B). No difference was observed for the Ara h 8-specific cells with the aforementioned phenotypes between the two groups. These data demonstrated that a specific memory CD4 T cell response with a predominant effector-TH2 phenotype against Ara h 2 and pooled Ara h 1, 3, and 6, but not against Ara h 8, was related to an allergic response, and, conversely, a weak or an absence of a specific memory CD4 T cell response against Ara h 2 and Ara h 1, 3, and 6 was related to an absence of a clinical response in sNPA subjects (Figures 4 and 5).

### Validation of the Link Between the Ara h Component-Specific TH2 CD4 T Cell Responses and Peanut Allergy

**Figure 6:**
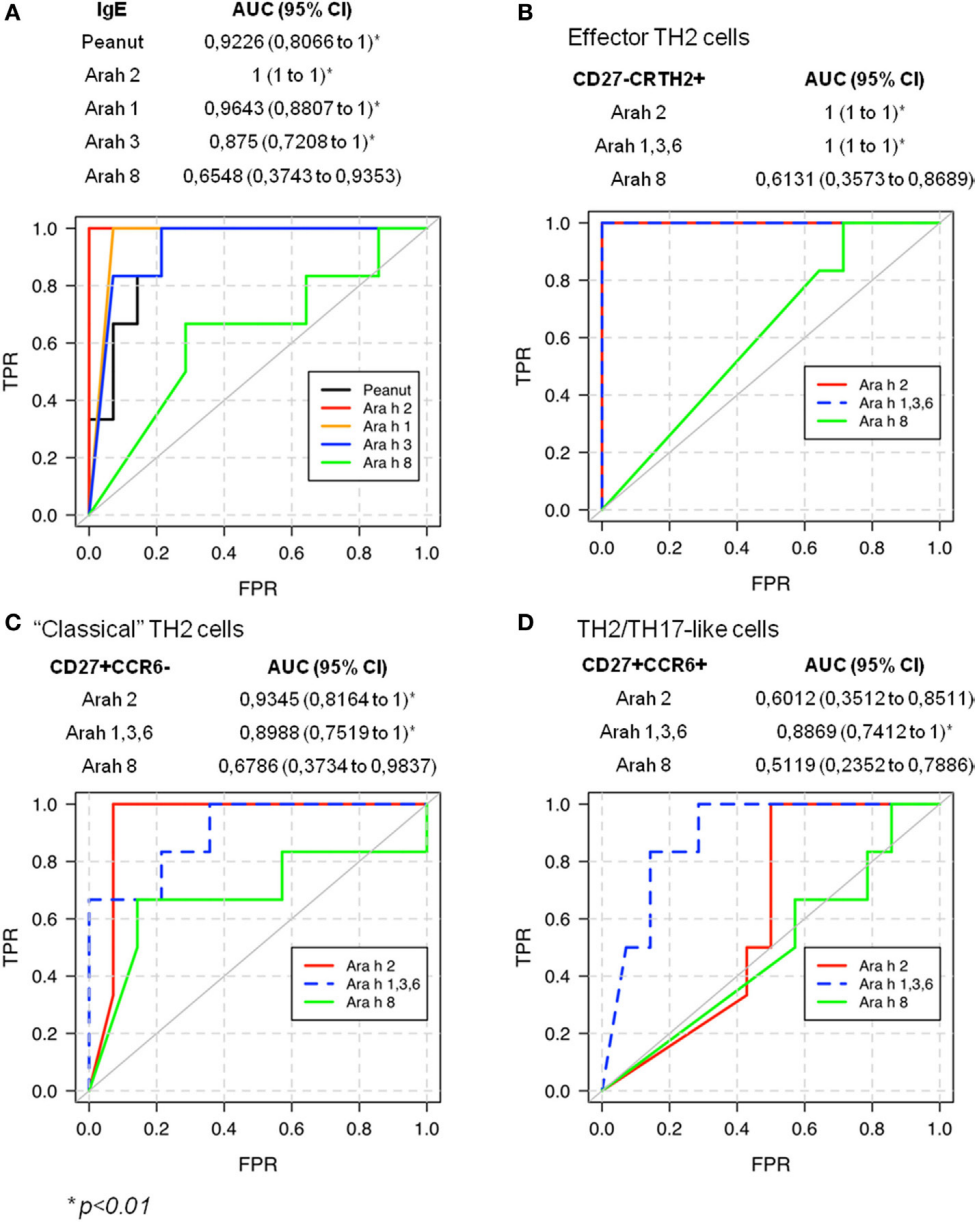
Receiver operating characteristic (ROC) validates allergic response for peanut on sensitized subjects.

Previous studies established that Ara h 2-specific IgE gave better predicting value of peanut allergy compared with whole peanut extract and other Ara h components (3–5). We observed a similar outcome in our cohort of PA and sNPA subjects (Figure 6A). The relevance of the Ara h component-specific CD4 TH2 cell responses in “predicting” peanut allergy was also evaluated by using receiver operating characteristic (ROC) analysis. Frequencies of different subsets of Ara h 2-, Ara h 1, 3, and 6-, and Ara h 8-reactive CD4 T cells after Ara h peptide stimulation were used to discriminate between PA and sNPA. The data showed that frequencies of Ara h 2 and Ara h 1, 3, and 6-reactive effector-TH2 and “classical” TH2 cells as well as Ara h 1, 3, and 6-reactive TH2/TH17-like cells gave significant AUCs compared with random classification, which demonstrate that these parameters are specific of an peanut allergy (Figures 6B–D). Frequencies of effector-TH2-specific cells were most sensitive in differentiating subjects between the two groups. By using frequencies of Ara h 2- or Ara h 1, 3, and 6-reactive effector-TH2 cells with a cutoff value of 1.05 or 1.35 specific T cells per million CD4^+^ T cells, respectively, the test could discriminate the PA and sNPA subjects in our cohort with 100% sensitivity and specificity, which validate the fact that a Ara h-specific CD4 TH2 cell response is linked to peanut allergy.

## Discussion

In peanut allergy, the Ara h 2-specific IgE level seems to be the most relevant value in discriminating between allergic and tolerant subjects at this time (3–5). However, the associated Ara h component-specific CD4 T cell responses are not well described; which is of interest in the view to develop personalized immunotherapy by using peptides from Ara h components.

Peanut-specific T cell responses have been studied in PA and non-allergic patients using peanut extract (13–15, 17, 29, 30) or different Ara h recombinant proteins (12) to explore the T cell reactivity. Additional studies have described Ara h 1 and Ara h 2-specific CD4 T cell epitopes using peptides (16, 18, 19, 31, 32). However, all these studies only examined response to a single Ara h component or to peanut extract. A comparative analysis of different Ara h component-specific CD4 T cell responses in relationship to component-specific IgE responses in allergic subjects has not been established. In addition, specific T cell responses to different Ara h components in non-allergic subjects with low sensitization are not well characterized. In this study, T cell responses in PA subjects were mainly directed against Ara h 2 and Ara h 1, 3, and 6. T cell response to Ara h 8 was very weak or absence. Both Ara h 2- and Ara h 1, 3, and 6-specific T cell responses in PA subjects were dominated by an effector-TH2 profile (CCR4^+^CD27^−^CCR6^−^). These cells are probably identical to the pathogenic effector TH2, TH2a and pathogenic TH2 (Tpath2) cells as described in the literature (28, 33, 34), and are the terminal differentiated TH2 cells (27, 28). Some unique features of these terminal-differentiated TH2 cells are their expression of CD161 and ST2 (receptor for IL-33) (28, 33, 34). In addition, Wambre et al. also demonstrated that the majority of peanut-specific T cells did express CD161 and the TH2a cells in PA subjects have the ST2 transcript (28). Thus, we do expect that Ara h 1, 2, 3, and 6-reactive cells with the CCR4^+^CD27^−^CCR6^−^ phenotype also express CD161 and ST2. These effector-TH2 cells express higher level of IL4, IL5, and IL-9 compared with “classical” TH2 cells and thus could represent pathogenic cells mediating allergy through the IL-33–ST2 signaling pathway (28, 34). In addition, CCR4^+^CD27^+^CCR6^−^ Ara h-specific cells were also detected. These “classical” TH2 cells may be the precursors for the effector-TH2 cell and TH2/TH17 cells. TH2/TH17-like responses, as implicated by T cells with CCR4^+^CD27^+^CCR6^+^ phenotype (24), were also observed and were mainly associated with Ara h 1, 3, and 6. Thus, PA subjects with Ara h 1, 3, and 6 responses in the absence of an Ara h 2 responses may elicit a TH2/TH17-like response and can potentially have a different clinical manifestation of the disease compared with subject with only a TH2 response. Subjects with majority of their peanut-specific cells with TH2/TH17 phenotype may also response differently to oral immunotherapy. The observation that Ara 1, 3, and 6 were more prone to elicit a TH2/TH17-like response also suggested that the intrinsic nature of the allergen could influence differentiation of T helper cells. A caveat of this conclusion is the limited number of subjects being examined. A study with a larger PA cohort will strengthen the current conclusion.

In this study, we show that a single Ara h component-specific effector-TH2 cell response correlates with multiple Ara h-specific-IgE levels. These observations could be explained by the now understood correlations between peanut and Ara h 2, Ara h 1, and Ara h 3 IgE levels, which support the idea of an IgE cross-reactivity between Ara h 2 and Ara h 1 and Ara h 3 (35). Although T cell cross-reactivity between cashew, hazelnut, and/or pistachio was reported (36), there is no evidence of T cell epitopes conservation within Ara h components. Moreover, some subjects present significant Ara h component-specific T cell response but low IgE levels for those Ara h components (subjects 2, 11, and 12, Figure 5). Globally, the peanut-IgE level is linked to the Ara h 2, 1, 3, and 6, but not Ara h 8, specific TH2 cell reactivity in allergic subjects. Thus, monitoring allergen-specific T cell frequency at the component level is essential to fully comprehend the role of these cells during peanut allergy but also in view to develop relevant specific immunotherapy.

Both CD154 upregulation assay and tetramer staining assay were utilized in the current study. The specificity of this CD154 upregulation method was verified by the class II tetramer staining technology in a subset of subjects with Ara h 2 responses. First, a modified CD154 upregulation assay was used to identify Ara h 2-specific T cell epitopes, which was further confirmed by staining with Ara h 2-specific tetramers. Two dominant Ara h 2 epitopes associated with DR1501 and DR0901 were identified. Second, *ex vivo* tetramer staining for Ara h 2-specific T cells was compared with the CD154 assay. Indeed, similar phenotype using both tetramer technology and CD154 assay was observed. It will be important to characterize and validate Ara h 1, 3, and 6 dominant epitope by this CD154 epitope mapping approach given that a relatively high percentage of Ara h 1, 3, and 6-specific CD4 T cell express CCR6, which is potentially a producer of IL-17 as observed in other food allergy (24).

Current oral immunotherapy trials in peanut use peanut flour. As intact peanut allergens would cross link IgE and lead to allergic reaction, there is an interest in using T cell epitope peptides as vaccine to treat peanut allergy. The observation that different subjects show heterogeneous T cell responses toward different Ara h components has implication on the development of this T cell epitope peptide vaccine. It would be necessary to involve peptides derived from more than one Ara h component in the vaccine.

The sensitization of non-peanut-allergic subjects could be due to an IgE cross-reactivity between Ara h 8 and birch/alder IgE (7, 37), which induces a false positive skin prick test, or peanut-IgE reactivity. Indeed, correlation between Ara h 8 and Alder/Birch IgE levels was observed in our cohort (Alder IgE: Spearman *r* = 0.9684, *p* < 0.0001; Birch IgE: Spearman *r* = 0.9786, *p* < 0.0001). By using ROC analysis, the frequencies of Ara h 2 and 1, 3, and 6-reactive effector-TH2 cells were found to be useful in distinguish PA from sNPA subjects, such as previously observed with Ara h 2-IgE or Ara h 2 basophiles response. Thus, as the IgE Ara h (but not Ara h8) component reactivity the Ara h-specific T cell reactivity is linked to peanut allergy.

Overall, the study shows that TH2 type T cell responses in PA subjects are directed against Ara h 2 and/or Ara h 1, 3, and 6 and are dominated by an effector-TH2 phenotype. Peanut-specific classical TH2 and TH2/TH17 cells are also present. However, responses toward different Ara h components vary among PA subjects but were linked to the peanut-IgE level and to peanut allergy. sNPA subjects lack effector-TH2 type peanut-specific T cell response, and their sensitization is linked to IgE cross-reactivity with birch/alder pollen. Thus, Ara h component (but not Ara h 8)-specific T cell responses are clearly linked to the peanut allergy and need to be targeted to fully treat patients.

## Ethics Statement

This study was approved by Benaroya Research Institute Institutional Review Board, and written informed consent was obtained from all enrolled subjects.

## Author Contributions

AR and VB performed the experiments. MF, SC, and KN provided the samples. AR, ElizabethW, ErikW, and WK analyzed the data and generated the figures. AR and WK wrote the manuscript.

## Conflict of Interest Statement

EW has become recently employed by Celgene in Seattle. All of the other authors declare that the research was conducted in the absence of any commercial or financial relationships that could be construed as a potential conflict of interest.
